# Near-infrared spectroscopy for assessing the tissue oxygen extraction rate during sepsis: relationship with outcome

**DOI:** 10.1186/cc14223

**Published:** 2015-03-16

**Authors:** A Donati, E Damiani, C Scorcella, S Tondi, S Ciucani, P Pelaia

**Affiliations:** 1 Università Politecnica delle Marche, Ancona, Italy

## Introduction

Microcirculatory dysfunction impairs tissue oxygenation during sepsis. We applied near-infrared spectroscopy (NIRS) to evaluate the tissue oxygen extraction rate in sepsis and its relationship with outcome.

## Methods

A prospective observational study; 14 septic patients underwent NIRS monitoring (thenar eminence) with a vascular occlusion test (using a 40% StO_2_ target) at admission to the ICU. Healthy volunteers (*n *= 27) were studied as controls. The slope of the desaturation curve was assessed separately for the first (StO_2_ down1) and the last part (StO_2_ down2) of the curve and the difference between, Down2 - Down1, was calculated.

## Results

**Figure 1:**
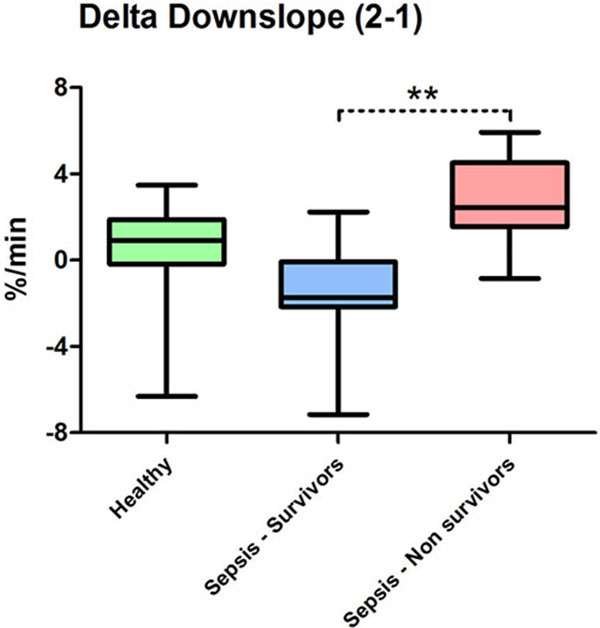


StO_2_ Down1 was lower in healthy volunteers as compared with septic patients (*P *< 0.05); no difference was seen between ICU survivors (*n *= 7) and nonsurvivors (*n *= 7). StO_2_ Down2 was similar between healthy volunteers and ICU survivors, while it was higher in nonsurvivors (*P *< 0.01 vs. healthy). ICU nonsurvivors showed higher Down2 - Down1 as compared with ICU survivors (*P *< 0.01, Figure 1).

## Conclusion

Tissue oxygen extraction was reduced in septic patients. Nonsurvivors showed a flattening in the last part of the desaturation curve during a VOT, while the first part of the StO_2_ downslope did not show any difference between survivors and nonsurvivors. This may reflect a tissue hypometabolic status, which may be better elicited in the final part of the ischemic challenge.

